# First record of *Zaitzevia* Champion, 1923 (Coleoptera, Elmidae) from Fujian Province, China, with descriptions of two new species

**DOI:** 10.3897/zookeys.1293.196561

**Published:** 2026-09-29

**Authors:** Dong-Ju Bian, Manfred A. Jäch

**Affiliations:** 1 CAS Key Laboratory of Forest Ecology and Silviculture, Institute of Applied Ecology, Chinese Academy of Sciences, Shenyang, 110016, China CAS Key Laboratory of Forest Ecology and Silviculture, Institute of Applied Ecology, Chinese Academy of Sciences Shenyang China; 2 Natural History Museum Vienna, Burgring 7, 1010 Vienna, Austria Natural History Museum Vienna Vienna Austria

**Keywords:** Macronychini, riffle beetles, taxonomy

## Abstract

Two new species of the riffle beetle genus *Zaitzevia* Champion, 1923 are described from Fujian Province, southeastern China: *Z. longyanensis***sp. nov**. and *Z. yanfengi***sp. nov**. The genus *Zaitzevia* is here recorded from Fujian for the first time. Photographs of diagnostic characters are provided.

## Introduction

The genus *Zaitzevia* Champion, 1923 currently comprises 36 valid species distributed in Asia and North America (Jäch et al. 2016; Jiang and Wang 2020, 2021; Bian and Zhang 2022; Iwata et al. 2022; Jiang and Chen 2023; Bian and Hu 2024; Peng et al. 2024; Bian and Jäch 2025; Jiang et al. 2025).

Currently, 23 species of the genus *Zaitzevia* are known from China, 19 of which have been described between 2020 and 2025 (Jiang and Wang 2020, 2021; Bian and Zhang 2022; Jiang and Chen 2023; Peng et al. 2024; Bian and Jäch 2025; Jiang et al. 2025). Nevertheless, there still exist numerous undescribed Chinese species of *Zaitzevia*, many of which are deposited in the Natural History Museum Vienna (Austria).

Two new species of *Zaitzevia* from Fujian Province are described below. The genus *Zaitzevia* is here recorded from Fujian for the first time. The remaining new species collected in Fujian during the China Water Beetle Survey (CWBS) in 1997 shall be described in forthcoming contributions.

## Material and methods

This study was based on a total of 24 specimens collected in 1997 and 2013. The holotypes of the new species and seven paratypes are deposited in the Institute of Applied Ecology, Chinese Academy of Sciences, Shenyang, China (**IAECAS**), while 15 paratypes are housed in the Natural History Museum Vienna, Austria (**NMW**).

With the exception of eight specimens, the material treated herein was collected from three localities during the China Water Beetle Survey (**CWBS**, see Jäch and Ji 1998):

CWBS loc. 252: NW Fujian Province; Nanping City Region [historical Shaowu Prefecture; erroneously transcribed as “Jianyuan Prefecture” in Jäch and Ji (1998)]; Guangze County (= Shuanxi); 12 km S Zhima Town and 2 km N Li Fang Village; small stream flowing from forested mountains (broadleaf trees, bamboo and *Cunninghamia*), ca 1 m wide, partly shaded by bushes, pools (filled with sand) and sections with granitic rocks and coarse crystalline gravel, 400 m a.s.l.; 22.I.1997; leg. H. Schönmann, L. Ji & M. Wang.

CWBS loc. 256: NW Fujian Province; Sanming City Region [historical Yanping Prefecture; erroneously transcribed as “Jianyuan Prefecture” in Jäch and Ji (1998)]; ca 20 km SE Yong’an City, 5 km SW Xiyang Village; small stream descending from Ziyungdong Shan (summit: 1629 m a.s.l.), fast flowing, <0.5 m wide, steep slopes with bushes, big granitic rocks, 550 m a.s.l.; 25.I.1997; leg. H. Schönmann, L. Ji & M. Wang.

CWBS loc. 263: Fujian Province; Longyan City Region; 2 km N Xiaochi Village, ca 20 km W Longyan City; mountain stream, 3–5 m wide, flowing through densely forested (broadleaf trees) steep valley descending from Meihua Shan, small waterfalls, big pools, crystalline sand and gravel, shaded, pools with decaying organic matter, 650 m a.s.l.; 30.I.1997; leg. H. Schönmann, L. Ji & M. Wang (see Jäch and Ji 1998: figs 14, 16).

Specimens were examined with a Leica M205 C stereomicroscope and an Olympus BX51 compound microscope. Male genitalia were placed in concentrated lactic acid in a cavity slide for at least several hours before they were examined. Photographs of habitus and genitalia were made with KEYENCE VHX2000 – Super Resolution Digital Microscope System. The first strial interval refers to the sutural interval.

Label data are cited verbatim. Lines of labels are separated by a backslash (“\”); different labels are separated by two straight lines (“ǀǀ”).

The following abbreviations are used in the text: **BL**—body length (= PL+EL); **BW**—maximum body width (= EW); **EL**—elytral length; **EW**—maximum elytral width; **PL**—pronotal length; **PW**—maximum pronotal width.

## Taxonomy

### Genus Zaitzevia

Taxon classificationAnimaliaColeopteraElmidae

Champion, 1923

3550ACF7-A002-51E4-B6CB-FADE9CA8E985

Zaitzevia Champion, 1923: 170. Type species: Zaitzevia solidicornis Champion, 1923. Awadoronus Kôno, 1934: 127 (synonymized by Nomura 1958). Type species: Awadoronus awanus Kôno, 1934.

### Zaitzevia longyanensis sp. nov.

Taxon classificationAnimaliaColeopteraElmidae

0071E183-D218-552D-AA0A-4D1C4926F259

https://zoobank.org/76AEA538-CBD6-4D2E-91B4-CFBBFCAFE1F3

Figs 1, 2

#### Type material.

***Holotype*** ♂ (IAECAS): • “China: Longyan City, 2 Km N \ Xiaochi Village ǀǀ 650 m, 1997.01.30 \ Leg. Ji & Wang (CWBS 263)”. Note: This locality is identical with the type locality of *Grouvellinus acutus* Bian & Jäch, 2018 (Coleoptera: Elmidae). ***Paratypes***: • 14 unsexed exs. (NMW) from the type locality: “China: Fujian, Longyan \ Xiaochi (20 km W Longyan) \ Meihua Shan, 650 m, 30.1.1997 \ leg. Ji & Wang (CWBS 263)”; • 1 unsexed ex. (NMW): “China: Fujian, Guangze \ Wuyi Shan, 12 km S Zhima \ Li Fang, 400 m, 22.1.1997 \ leg. Ji & Wang (CWBS 252)”.

**Figure 1.**
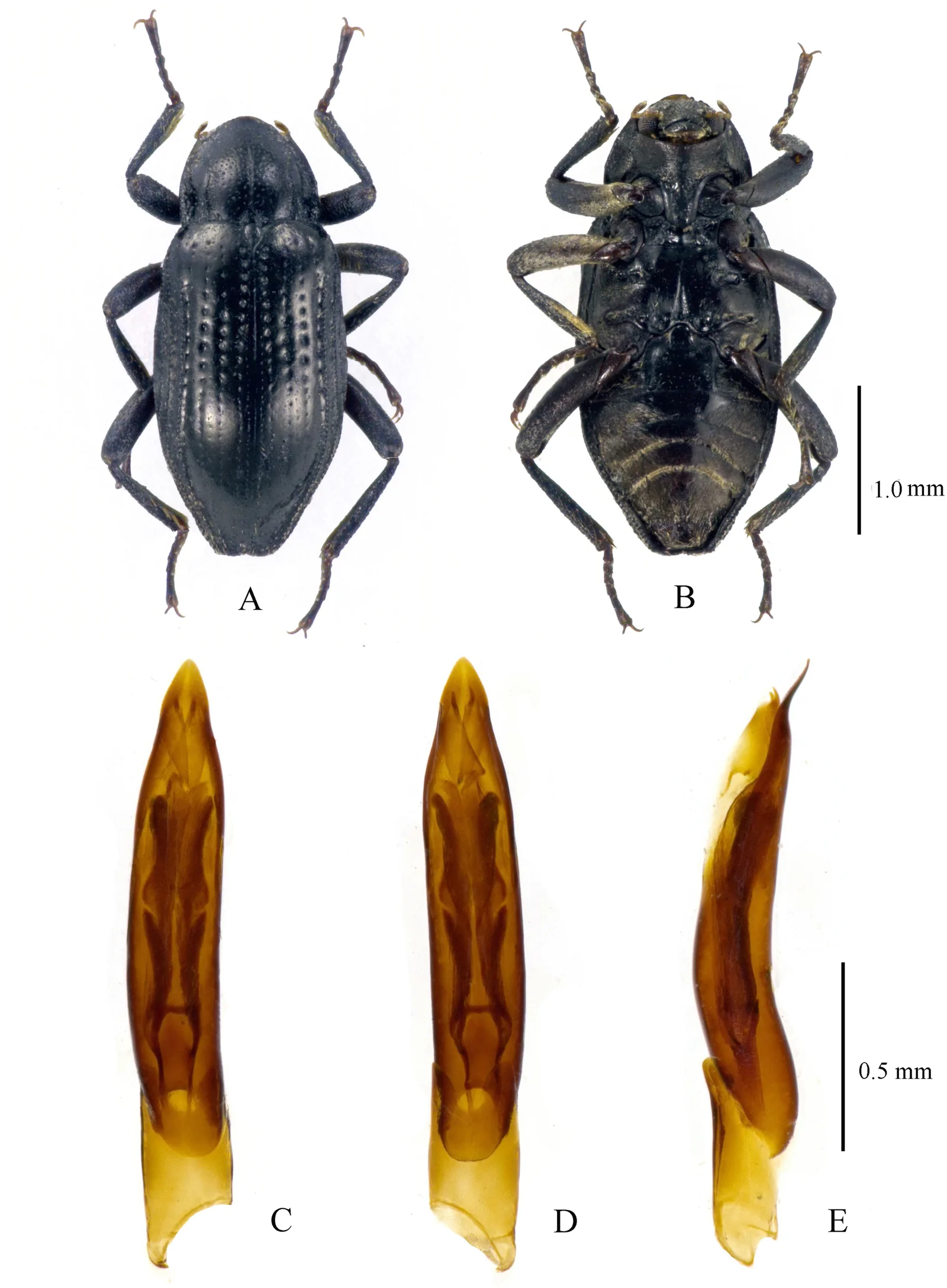
*Zaitzevia longyanensis* sp. nov., holotype, male. **A, B**. Habitus in (A) dorsal and (B) ventral view; **C–E**. Aedeagus in (**C**) ventral, (**D**) dorsal and (**E**) lateral view.

**Figure 2.**
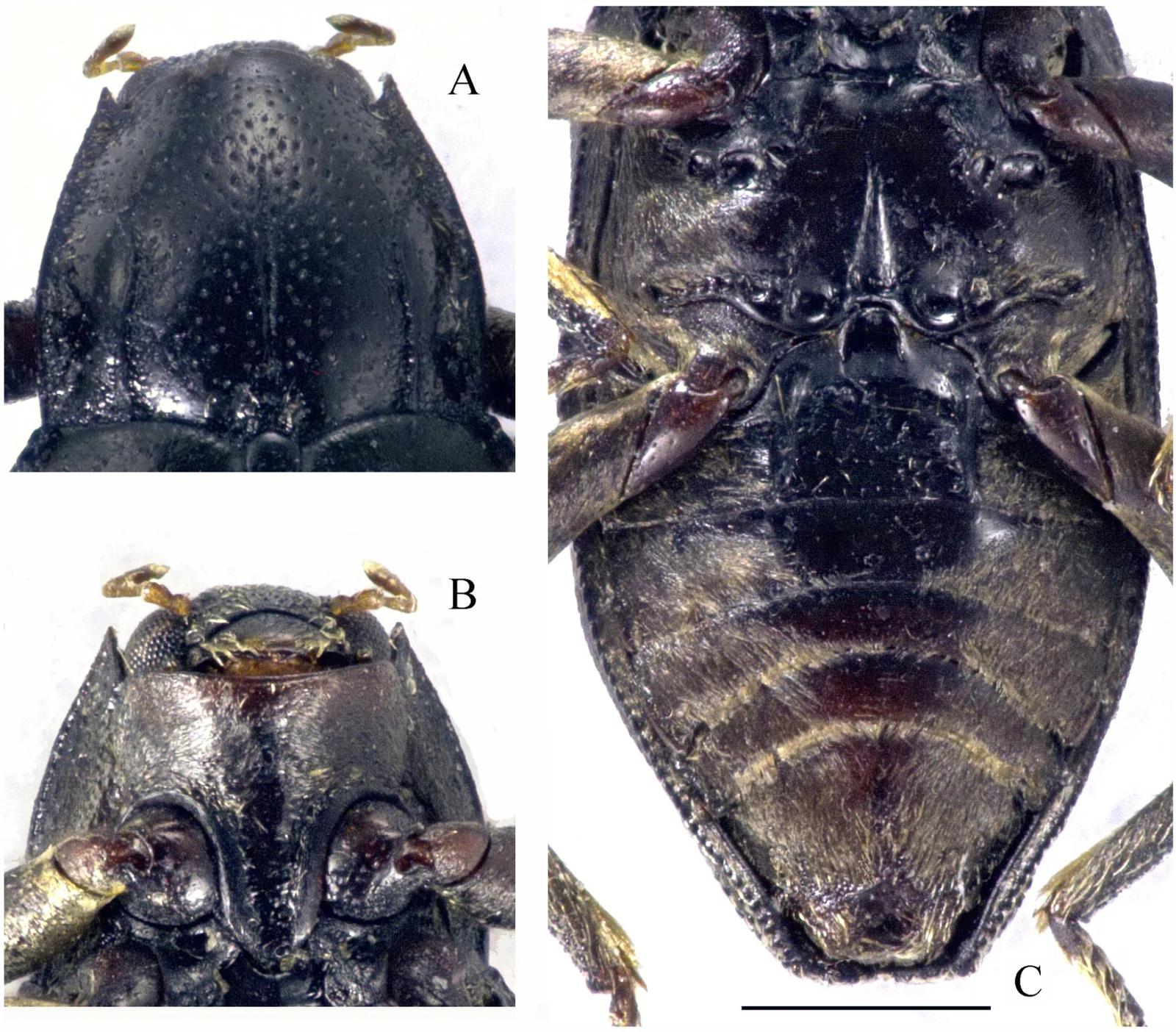
*Zaitzevia longyanensis* sp. nov., holotype, male. **A**. Pronotum; **B**. Prosternum and prosternal process; **C**. Metaventrite and abdominal ventrites. Scale bar: 0.5 mm.

#### Diagnosis.

*Zaitzevia longyanensis* can be distinguished from all other Chinese species of the genus by the combination of the following characters: body size 2.64–2.90 mm, body elongated obovate, anterior pronotal corners strongly produced and acute, elytral stria I distinctly punctate in basal half, punctures on striae II–IV very large from about basal 0.2–0.5, but more or less obsolete in basal 0.2 and distal 0.5, and elytral apex subtruncate. We are not aware of any described species which might be confused with *Z. longyanensis* based on external characters. Regarding male genitalia, *Z. longyanensis* vaguely resembles *Z. fodingshana* Jiang & Chen, 2025 from Guizhou Province, but it can be distinguished from the latter by the apex of the median lobe being robust in ventral view, by the sclerotizations of the endophallus being well developed from basal 0.10 to distal 0.25, somewhat resembling a bowknot, while in the latter, the apex of the median lobe is slenderer and more acute in ventral view, the slerotizations of the endophallus are rod-like and well developed from subbase to distal 0.35; externally, *Z. fodingshana* differs significantly from the new species, especially in the much less produced anterior pronotal corners and in the rounded elytral apices.

#### Description.

**Male** (holotype). BL 2.90 mm, BW 1.30 mm. Body elongate obovate (Fig. 1A). ***Colour***. Dorsum black, ventral side (Figs 1B, 2B, 2C) dark brown to black, tarsi brown, antennae yellowish-brown.

***Head***. Labrum transverse, surface smooth and shiny, sparsely punctate and pubescent, anterior margin convex, not emarginate medially, lateral margin with long setal fringe.

***Pronotum***. PL 0.75 mm, PW 0.93 mm. Pronotum broadest at basal 0.4, slightly narrowed posteriorly, and distinctly attenuate anteriorly, surface smooth and shiny, densely punctate and sparsely pubescent. Anterior corners distinctly produced and sharply pointed, posterior corners almost rectangular. Anterior margin slightly produced anteriad. Median groove narrow and shallow, present in basal 0.1–0.6. Base with two admedian pairs of small granules in front of scutellum. Sublateral carinae extending from base to basal 0.4, distinct, subparallel in basal 0.3, then curved inward, mesally bordered by a groove.

***Elytra***. EL 2.15 mm, EW 1.30 mm. Elytra subparallel in basal 2/3, distinctly attenuate in distal 1/3, apex subtruncate. Stria I distinctly punctate in basal half, but more or less impunctate in distal half. Punctures on striae II–IV very large in about basal 0.2–0.5, but more or less obsolete in basal 0.2 and distal 0.5. Intervals smooth and shiny, intervals I and III each furnished with a row of minute setiferous punctures. Carinae on intervals V extending from about basal 0.15 to the apex; carinae on intervals VI–VII extending from base to apex.

***Ventral side of thorax***. Prosternum densely punctate and granulate, with dense yellowish plastron anterior of procoxae (except along midline). Prosternal process unpubescent, gradually narrowed, more or less shiny, with some larger punctures, apex broadly rounded, lateral margin distinctly rimmed. Metaventrite broadly impressed in posterior 0.8, disc smooth and shiny, only with a few small punctures, not pubescent; median groove situated in posterior 0.8, gradually narrowing anteriad, with a pair of very large, admedian basal pits; lateral areas covered by dense plastron, with two transverse rows of large punctures, one behind mesocoxa and one anterior of metacoxa.

**Legs**. Without distinct sexual dimorphism, largely covered by plastron.

***Ventrites***. Disc of ventrite I smooth and shiny, with widely spaced fine punctures, admedian carinae strongly developed. Middle of ventrites II–IV, and middle of basal 1/3 of ventrite V smooth and shiny, only with a few fine punctures; lateral parts of ventrites I–IV and lateral parts of ventrite V in anterior 0.4 covered by plastron; distal 0.6 of ventrite V partly punctate and granulate, covered with setae that become increasingly longer towards produced and pointed posterior corners; apex of ventrite V rather distinctly emarginate, concavely impressed and unpubescent between acute corners, which are slightly bent ventrad.

***Aedeagus***. 1.6 mm long. Median lobe about 2.4 times as long as phallobase, slightly broadened in basal 1/4, then subparallel to distal 1/4, distinctly narrowed in distal 1/4, with a pair of small subapical teeth at apical end of ventral sac; in lateral view, median lobe distinctly curved dorsad apically. Sclerotizations of endophallus well developed, somewhat resembling a bowknot. Parameres not discernible.

**Female**. Externally, females cannot be easily distinguished from males. On average, posterior part of metaventrite and median groove less distinctly impressed in females. Apical corners of ventrite V only hardly noticeably produced in females, and less strongly impressed between corners.

#### Variability.

BL varies from 2.64–2.90 mm. The median pronotal groove varies in length and width, in some specimens reaching only from basal 0.2–0.6. The small admedian granules located at the base of the pronotum vary in size and number (2–6), being sometimes quite distinctly developed, but sometimes hardly visible. As in many species of *Zaitzevia*, the size and number of punctures on the elytral disc varies considerably.

#### Distribution.

China: Nanping and Longyan city regions in northwestern and southwestern Fujian Province.

#### Etymology.

This specific epithet is used as an adjective and refers to Longyan City, the region of the type locality.

### Zaitzevia yanfengi sp. nov.

Taxon classificationAnimaliaColeopteraElmidae

1EF500D6-5009-5BE6-8987-2FB56AF4E6DF

https://zoobank.org/53499BF8-8AEA-41FE-920B-CD00BE48D20D

Figs 3, 4

#### Type material.

***Holotype*** ♂ (IAECAS): • “China: Fujian, \ Yong’ an City, \ Shangping Vill. ǀǀ 25°57'27.246"N, \ 117°30'46.848"E \ 543 m, 2013.12.15 \ leg. Tong”. ***Paratypes***: • 3 ♂♂, 4 ♀♀, same data as holotype.

**Figure 3.**
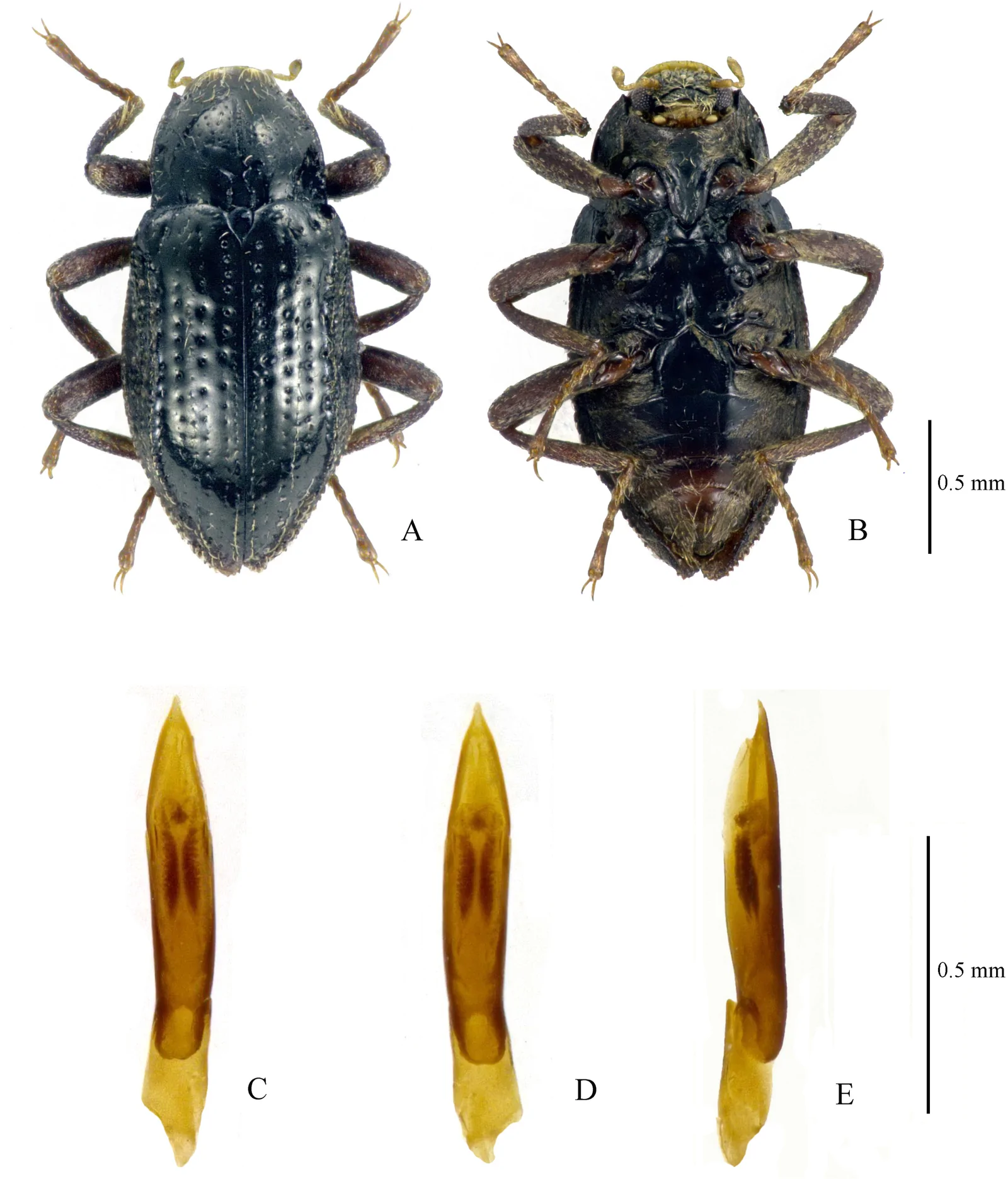
*Zaitzevia yanfengi* sp. nov., holotype, male. **A, B**. Habitus in (**A**) dorsal and (**B**) ventral view; **C–E**. Aedeagus in (**C**) ventral, (**D**) dorsal and (**E**) lateral view.

**Figure 4.**
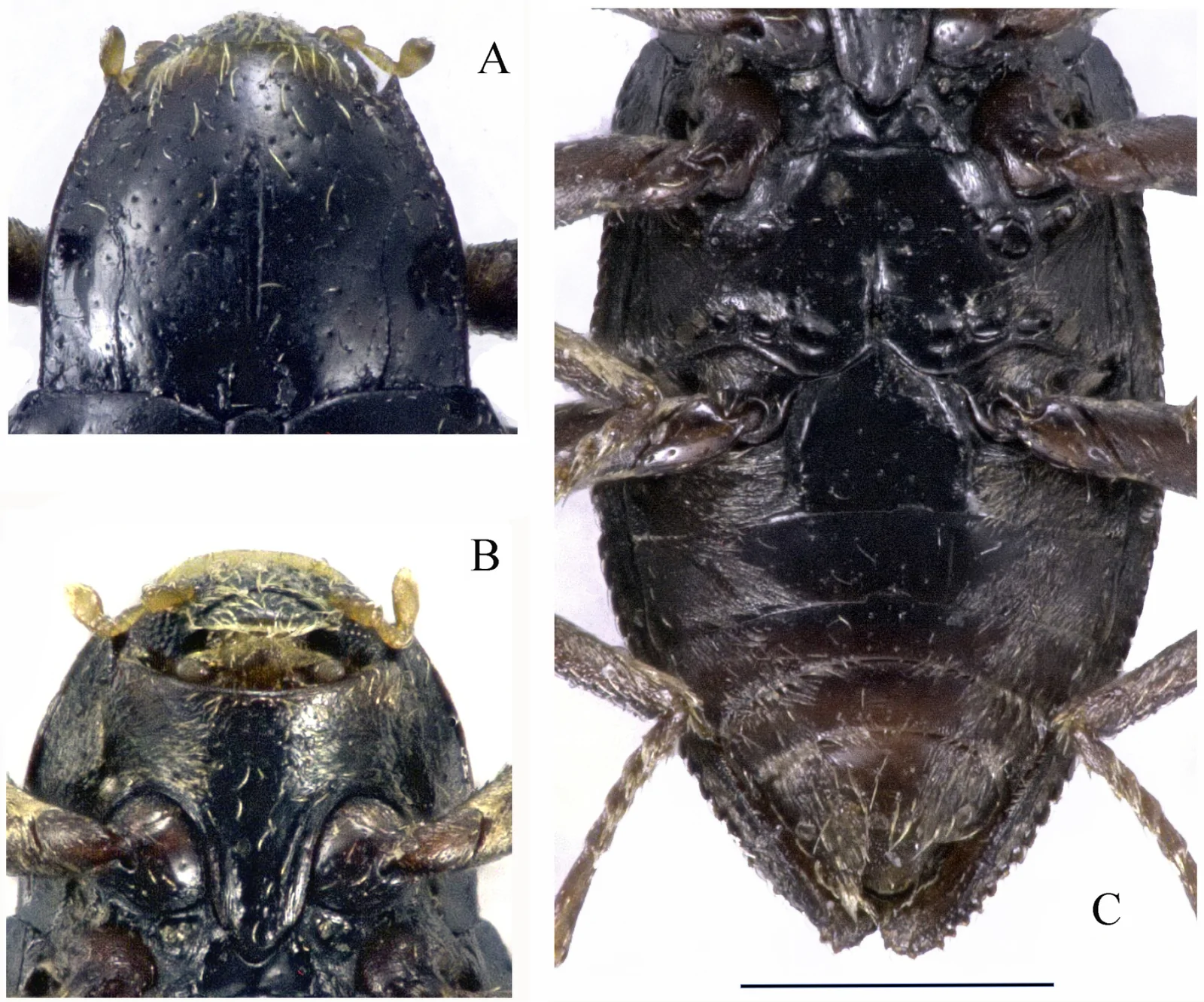
*Zaitzevia yanfengi* sp. nov., holotype, male. **A**. Pronotum; **B**. Prosternum and prosternal process; **C**. Metaventrite and abdominal ventrites Scale bar: 0.5 mm.

#### Diagnosis.

Externally, the new species somewhat resembles *Zaitzevia acuta* Bian & Hu, 2024 and *Z. nanlingensis* Bian & Hu, 2024 from Guangdong Province, especially by the acute anterior pronotal corners and by the unevenly distributed elytral punctures, but it can be easily distinguished from *Z. acuta* by the distal 0.4 of penis being less sharpened, by the endophallus possessing a pair of lanceolate sclerotized bands in basal 0.4–0.6, and by lacking subapical teeth at the apical end of the ventral sac, while in *Z. acuta*, the penis is distinctly sharpened in the distal 0.4, the endophallus possesses a pair of short oval sclerites in 0.4–0.5, and a pair of small reniform sclerites immediately posterior of the oval sclerites, and a pair of small teeth situated at the distal 1/6; it can be distinguished from *Z. nanlingensis* by the cuspidal apex of the penis, by the endophallus possessing a pair of lanceolate sclerotized bands in the basal 0.4–0.6, and lacking subapical teeth at the apical end of the ventral sac, while in *Z. nanlingensis*, the apex of the penis is narrowly rounded, and the endophallus possesses a prominent subcircular annular sclerite in the basal 0.6–0.7, a weakly sclerotized, helmet-shaped sclerite, and a pair of well-developed teeth at distal 1/6.

#### Description.

**Male** (holotype). BL 2.00 mm, BW 0.90 mm. Body elongated oval (Fig. 3A). ***Colour***. Dorsum black, ventral side brown to black (Figs 3B, 4B, 4C), legs except tarsi brown, antennae and tarsi yellowish brown.

***Head***. Labrum smooth and shiny, densely pubescent and punctate, anterior margin not emarginate, broadly rounded, with long and dense setae laterally. Clypeus smooth and shiny, densely pubescent and sparsely granulate. Frons densely pubescent and granulate.

***Pronotum***. PL 0.53 mm, PW 0.70 mm. Subparallel in basal 0.4, then gradually attenuate anteriorly. Anterior margin slightly produced anteriad. Anterior corners produced and very sharp, posterior corners almost rectangular. Surface smooth and shiny, fine punctures densely distributed. Pubescence dense in anterior half, but sparse in basal half. Median groove narrow and shallow, extending from basal 0.2–0.6. Sublateral carinae moderately strong, present in basal 0.4, subparallel in basal 0.2, then bent inwards, groove indistinct. Base with three pairs of granules in front of anterior corners of scutellum.

***Elytra***. EL 1.47 mm, EW 0.90 mm. Broadest near middle, slightly narrowed anteriorly and distinctly attenuate posteriorly; apices separately rounded and subacute. Stria I distinctly punctate in basal half, but more or less impunctate in distal half. Punctures on striae II–IV distinct and of unequal size from about basal 0.2–0.6, but more or less obsolete in basal 0.2 and distal 0.4. Intervals smooth and shiny, intervals I and III with a row of small setal punctures. Intervals II and IV almost without any punctures. Carinae on intervals V extending from basal 0.1 to the apex, carinae on intervals VI and VII complete.

***Ventral side of thorax***. Prosternal process gradually narrowed in basal 0.9, distinctly narrowed in distal 0.1, apex narrowly rounded; lateral margin distinctly rimmed; disc smooth and shiny, only with a few punctures and setae. Metaventrite broadly impressed in posterior 0.8, disc smooth and shiny, with only few punctures, glabrous; median groove deep, present in posterior 0.6; with two transverse rows of large punctures, one behind mesocoxa and one anterior of metacoxa; lateral parts of metaventrite covered by dense plastron.

***Legs***. Without distinct sexual dimorphism, largely covered by plastron.

***Ventrites***. Disc of ventrite I smooth and shiny, with widely spaced fine punctures, admedian carinae well developed. Middle of ventrites II–IV, and middle of basal 1/3 of ventrite V smooth and shiny, only with a few fine punctures; lateral parts of ventrites I–IV and lateral parts of ventrite V in anterior 0.4 covered by plastron; distal 0.6 of ventrite V partly punctate and granulate, covered with setae that become increasingly longer towards posterior corners; apex of ventrite V more or less subtruncate, corners hardly produced.

***Aedeagus***. 0.82 mm long. Median lobe about 2.2 times as long as phallobase, slightly broadened from base to basal 0.2, then subparallel to basal 0.6, distinctly acuminate in distal 0.4. Endophallus with a pair of lanceolate sclerotized bands in basal 0.4–0.6. Remnants of parameral apices visible at about apical 0.3 of median lobe.

**Female**. Similar to males, but metaventrite slightly convex, not impressed. Due to some variability, ventrite V not distinctly different from male; in some males, the lateral margin of ventrite V is provided with a dense row of very small spines, that obviously have been (partly) rubbed off in some specimens; we did not observe such spines in the females.

#### Variability.

BL varies from 1.8–2.1 mm. The median pronotal groove varies slightly in length. As in many species of *Zaitzevia*, the number, size, and distance of the punctures on the elytral disc are subject to some variability. Elytral apices separately or conjointly rounded.

#### Comparative remarks.

We would like to point out that a very similar but obviously different species has been collected in the Nanping, Sanming and Longyan city regions in western Fujian Province (CWBS 252, 256, 263). These 11 specimens deposited in the NMW differ from the type material of *Zaitzevia yanfengi* sp. nov. in the following characters: 1) BL 1.54–1.60 mm, BW 0.68–0.71; 2) anterior pronotal corners thinner and even more strongly acuminate; 3) sublateral pronotal carinae more prominent, adjacent groove distinct; 4) shape of elytra more elongate; 5) aedeagus 0.78 mm long, lanceolate bands in basal 0.4–0.6 of endophallus poorly sclerotized; apical fourth of median lobe with thin longitudinal sclerotized rod, which branches into an inconspicuous claw-like structure in the apical part of the ventral sac. At this stage we refrain from a formal description and prefer to wait until more material and hopefully also molecular data will become available to confirm the distinctness of these 11 specimens.

#### Distribution.

China: Sanming City Region in central western Fujian Province.

#### Etymology.

The specific epithet is a noun in the genitive case honoring Yanfeng Tong (Shenyang, China), who collected the type series.

## Supplementary Material

XML Treatment for Zaitzevia

XML Treatment for Zaitzevia longyanensis

XML Treatment for Zaitzevia yanfengi

## Additional information

### Artificial Intelligence (AI) use

The authors accept full responsibility for the content of the manuscript, including the disclosure of any use of AI.

No AI tools were used in the preparation of this manuscript.

### Author ORCIDs

D.-J. Bian https://orcid.org/0000-0002-9324-8026

M.A. Jäch https://orcid.org/0000-0002-2990-3604

### Data availability

All of the data that support the findings of this study are available in the main text.
